# Adolescent social media use and psychiatric outcomes: a longitudinal mediation analysis via interpersonal distrust, sleep, and self-image

**DOI:** 10.1007/s00127-025-02999-w

**Published:** 2025-09-29

**Authors:** Dimitris I. Tsomokos

**Affiliations:** Department of Psychology and Human Development, UCL Institute of Education, University College London, London, UK

**Keywords:** Self-harm, Suicidality, Mood disorders, Trust, Social media, Adolescence

## Abstract

**Purpose:**

The present study investigated the longitudinal associations between social media use (SMU) in early adolescence (age 11) and psychiatric outcomes (age 17) via interpersonal distrust, later bedtime, and negative self-image (age 14) after controlling for prior mental health (age 7) and a range of confounders.

**Methods:**

A structural equation model linked SMU to psychological distress (Kessler-6) via distrust, time-to-sleep, and negative self-perception, using data from a birth cohort in the United Kingdom. From 12,732 eligible adolescents at age 11 (interviewed January 2012 to February 2013), 8,913 participants (52% female, 18% non-White) had complete data on exposure and outcome, thus included in the analytic sample. Sex-stratified analyses were performed, as well as secondary outcome analyses for internalizing/externalizing problems, and a latent variable of ‘psychiatric problems’ (depression/anxiety diagnosis, self-harm, suicidality).

**Results:**

There were significant indirect paths through distrust (standardized $\:\alpha\:\beta\:=0.004,\:p=.02$), later time-to-sleep ($\:0.010,\:p<.001$), and negative self-perception ($\:0.012,\:p<.001$) after adjustments. The path through distrust was significant for females but not for males, whereas the two other indirect paths were significant for both males and females (this also held true for internalizing, externalizing, and psychiatric problems). Use of self-report measures and a lack of detailed information on the nature of SMU limit these findings.

**Conclusion:**

Social media use is prospectively associated with psychiatric symptoms in adolescence to the extent that it fosters interpersonal distrust, delays bedtime, and degrades self-image, especially for females. Interventions aimed at promoting trust and belonging, good sleep hygiene, and positive self-image, should be considered from a public health perspective.

## Introduction

Adolescent mental health has emerged as a major public health concern around the world [1–3], and it is now recognized that early to middle adolescence is a critical age of onset for a wide range of psychiatric disorders [4–6]. To understand the multitude of factors that may contribute to reported increases in prevalence rates [7], the relationship between social media use (SMU) and youth mental health has been examined in some detail [8–14], with several reviews and meta-analyses appearing on the subject in recent years [15–28]. Although many aspects of the mechanisms involved in this relationship remain unclear [29, 30], the emerging consensus is that there is a small—to potentially moderate—association between SMU and adolescent psychopathology [31], which appears to be stronger for females. So far, however, most research has been cross-sectional with moderate-sized samples, focusing on late adolescence.

Despite some progress on longitudinal research [32–38], there remain several gaps in the literature. First, there is a lack of large-scale longitudinal studies in which the time interval between SMU and psychiatric outcomes is longer than a few months to a year, as we cannot rule out the possibility that the association between SMU and mental health persists over longer timeframes during adolescence, or that some of the mechanisms involved might take longer to affect outcomes. Second, the pathways through which early exposure to social media impacts later mental health probably extend far beyond what is currently known or hypothesized about the mediating roles of sleep, social comparison, and self-perception of physical appearance [29]. Third, sex differences [37] in such long-term processes remain largely unexplored in the context of longitudinal studies.

In terms of known biopsychosocial mechanisms in the association between social media use and mental health difficulties, sleep has emerged as an important factor [39–41]. In a systematic review of forty-two studies in youth aged 16 to 25 years [42], higher frequency of social media use was associated with both poorer sleep quality and negative mental health outcomes, both cross-sectionally (thirty-six studies) and longitudinally (six studies). One of the strongest predictors of sleep quality is sleep duration, which is negatively associated with later bedtimes in adolescence [43–45]. Another key factor appears to be negative self-perception and body image [46–50]. Experimental and longitudinal studies have provided evidence of a causal relationship between the consumption of appearance-ideal images in social media and body image disturbance [51]. However, most of the experimental studies were performed on adults, and longitudinal data on children and adolescents remains scarce.

Furthermore, recent studies have provided evidence for a link between depression and interpersonal distrust in middle adolescence, both cross-sectionally [52] and longitudinally [53] (ages 14 to 17). The link between distrust and psychopathology [54, 55] has been interpreted through the lens of Social Safety Theory [56–58], which considers generalized trust perceptions as an index of positive social safety schemas [59], namely, perceptions of social support, belonging, and acceptance, whereas generalized distrust indexes negative social safety schemas reflecting perceptions of social isolation, rejection, and a lack of perceived social support. The former is conducive to human health and development, while the latter—interpersonal distrust and perceptions of social isolation—are harmful to both physical and mental health through complex pathways that involve neuroendocrinological systems and persistent inflammatory responses [56, 57]. However, the role of social media use in the formation of maladaptive social safety schemas (e.g. indexed via interpersonal distrust [53]) has not been addressed in any age group, even though there is mixed evidence on the association between social media use and perceived social support in adults [60–63].

The present research addresses these gaps by considering a large-scale, longitudinal, nationally representative birth cohort survey from the United Kingdom. The Millennium Cohort Study was designed to track the development of children born from late 2000 to early 2002, drawn from around 19,000 families across the four UK countries (England, Scotland, Wales, and Northern Ireland). The cohort belongs to what typically has been referred to as Generation Z[64]*Z*^64^. The majority of cohort members turned 11 years old during 2012, when online access to social media began to rise in the UK, the United States, and elsewhere [65]. During that period (from January 2012 to February 2013), the fifth wave of the Millennium Cohort Study survey took place, and participants were asked: “How often do you visit a social networking website on the internet, such as Facebook or Bebo?” Despite this being a single questionnaire item that does not provide any insight into the type of SMU [38] (e.g. active or passive use), it nevertheless allows us to infer the frequency of exposure to social networks at that time, and to consider it as a proxy for SMU in early adolescence more broadly.

Therefore, the main hypothesis in the present work was that SMU at the cusp of adolescence (age 11) for Generation Z was prospectively associated with psychological distress—measured by the short Kessler scale—in late adolescence (age 17), and that the development of interpersonal distrust between the two timepoints (at age 14) mediated this association along with poorer sleep (later bedtimes) [22, 36] and negative self-perception of physical appearance [10, 33]. Confounders related to both exposure and outcome (or mediators and outcome) included biological sex [37], race and ethnicity [7], neighborhood disadvantage and household income [14], maternal education and maternal mental health [6], as well as the participants’ prior mental health difficulties in childhood [31] (at age 7).

## Methods

The Millennium Cohort Study follows the lives of more than 19,000 children born between late 2000 and early 2002 in the United Kingdom, and fully anonymized datasets have been released in the public domain, corresponding to seven survey waves (at ages 9 months, and 3, 5, 7, 11, 14, 17 years) [66, 67]. As explained in more detail elsewhere [66, 68], a consortium of UK government stakeholders and the Economic and Social Research Council funded the survey, and extensive ethical approval processes were dictated in each wave by the National Health Service Ethics Committee system (the primary caregiver, which was the mother in the vast majority of cases, provided informed consent before any interviews took place in the child’s home, and children themselves provided their assent at the age 11 wave, and consent at age 14 and 17). The sampling frame for the survey was based on the UK’s electoral ward areas [67], and the survey weights used in the present study were such that participants living in disadvantaged areas (i.e., families whose residence was in neighborhoods with high levels of child poverty) were over-represented. In the age 11 wave, there were 12,732 singletons or first-born twins or triplets still participating in the survey. Of these, 8,913 participants (52% female, 17% non-White, 43% in disadvantaged areas) had complete records on both the exposure and the primary outcome and were included in the final sample.^1^

### Measures

*SMU (age 11)*: In the age 11 wave, participants were asked: “How often do you visit a social networking website on the internet, such as Facebook or Bebo?” The possible responses were: “Never” (value of 0); “Less often than once a month” (1); “At least once a month” (2); “At least once a week” (3); and “Most days” (4). A dichotomous version of this variable can be derived, with values “Not used” (corresponding to 0) and “Used” (for values > 0).

*Interpersonal distrust (age 14)*: At age 14 participants were asked: “On a scale from 0–10, where 0 means not at all and 10 means completely, how much would you say you trust other people?” In the present study, this measure of generalized interpersonal distrust is a numerical variable from 0 (*completely trusting others*) to 10 (*not at all trusting others*).

*Time-to-sleep* (age 14) was a numerical variable, from 1 to 5, derived from the self-reported time (within hour slots) of falling asleep on school nights. At the age 14 wave, participants were asked to select their usual time of falling asleep: (1) before 9 pm, (2) 9–9:59pm, (3) 10–10:59 pm, (4) 11–midnight, or (5) after midnight.

*Self-perception of physical appearance* (age 14) was a variable derived from an item in the Wellbeing Grid, which asked participants: “How do you feel about the way you look?” In our work, this was a numerical variable from 1 to 7 (in increments of 1), where 1 corresponds to completely happy with physical appearance, and 7 corresponds to not at all happy.

*Psychological distress (age 17):* Psychological distress (age 17): The primary outcome was the Kessler-6 scale [69], completed by cohort members who had participated in the age 17 wave (with Cronbach’s $\:\alpha\:=0.86$). The scale is composed of six items asking participants how they had been feeling over the last 30 days, scored from 0 (“none of the time”) to 4 (“all of the time”). As a result, the total score is from 0 to 24; higher scores indicated greater distress. A score of 13 or more is clinically relevant as it indicates the likely presence of (unspecified) psychological distress [69].

*Internalizing and externalizing problems (age 17):* The secondary outcomes used in additional analyses were derived from the Strengths and Difficulties Questionnaire [70], completed by cohort members at age 17. There were 20 items in all, with 5 questions in each subscale of mental health difficulties (emotion, peer, conduct, and hyperactivity or inattention), and the total score ranged from 0 to 40. A score of 17 or higher is clinically relevant as it may indicate the presence of mental health difficulties. The emotion and peer subscales combine into internalizing problems (Cronbach’s $\:{a}_{int}=.74$), while the conduct and hyperactivity/attention subscales combine into externalizing problems ($\:{a}_{ext}=.75$).

*Psychiatric problems (age 17)*: This latent variable was composed of three binary self-report items (yes/no): (a) whether the participant had received a clinical diagnosis of depression or anxiety by the time of the interview at age 17; (b) whether the participant self-harmed (cutting, stabbing, burning, bruising, or pinching themselves, or pulling out their hair); and, (c) whether the participant had ever attempted suicide (“Have you ever hurt yourself on purpose in an attempt to end your life?”).

*Confounders (ages 7–11).*

*Area disadvantage:* The social background of the cohort member was based on the sampling frame, which tracked neighborhood deprivation using the Child Poverty Index (an area’s relative disadvantage at the age 11 wave, determined by whether the neighborhood was in the upper quartile of the index, i.e., among the poorest 25% in each country). England also had an ethnic minority stratum, namely, areas in which at least 30% of their population fell into the broad categories of ‘Black’ (Black Caribbean, Black African and Black Other) or ‘Asian’ (Indian, Pakistani and Bangladeshi), as defined by the Census. *Sex* (male or female) was provided by the main respondent at the beginning of the survey (age 9 months). *Ethnicity* (White, Mixed, Indian, Pakistani and Bangladeshi, Black or Black British, Other Ethnic group including Chinese or Other) was a categorical variable based on the categories of the previous Census. The family’s *income* (age 11) was provided in OECD equivalised income quintiles. *Maternal education* (age 11) was a dichotomous variable (degree or no degree from a higher educational/vocational institution), based on National Vocational Qualifications. *Prior mental health difficulties* (for the cohort member at age 7) was a numerical variable, ranging from 0 to 40, given by the total score from the four difficulties subscales (emotion, peer, conduct, and hyperactivity/inattention) of the Strengths and Difficulties Questionnaire, completed by the child’s primary caregiver.

### Overall analytic plan

The main steps involve preliminary, core, and additional analyses [71]. First, we analyse the demographic characteristics of the sample, sample bias, pattens of missingness, correlations among the numerical variables, and plots of the raw data (e.g., boxplots, scatterplots, and alluvial plots). For brevity, only a fraction of this preliminary analysis is presented here, but details of all the steps involved and complete results of our R code are in the Supplemental Online Material (SOM) published on the Open Science Framework repository [71]. Second, the core analysis was performed using survey weights and structural regression modelling with various levels of adjustment, before and after data imputation (using multiple imputations via chained equations and following Rubin’s rules to combine them [71, 72]). For the direct link between SMU and psychiatric outcomes, binary exposure and outcome measures were also used (i.e., SMU exposure versus non-exposure, and presence of psychopathology based on a validated cut-off for the Kessler-6 scale) to calculate crude/adjusted odds ratios (adjustment with confounders but without the mediators, as these are on the ‘causal’ path). For these, depression/anxiety diagnosis, self-harm and suicidality were also considered separately. In two additional analyses, we considered the secondary outcomes (namely, internalizing and externalizing problems, and the latent variable of psychiatric problems). Finally, a sensitivity analysis was conducted, where the inclusion criteria for the analytic sample were changed (such that the exposure and focal mediator of distrust had valid records).

## Results

**Fig. 1:**
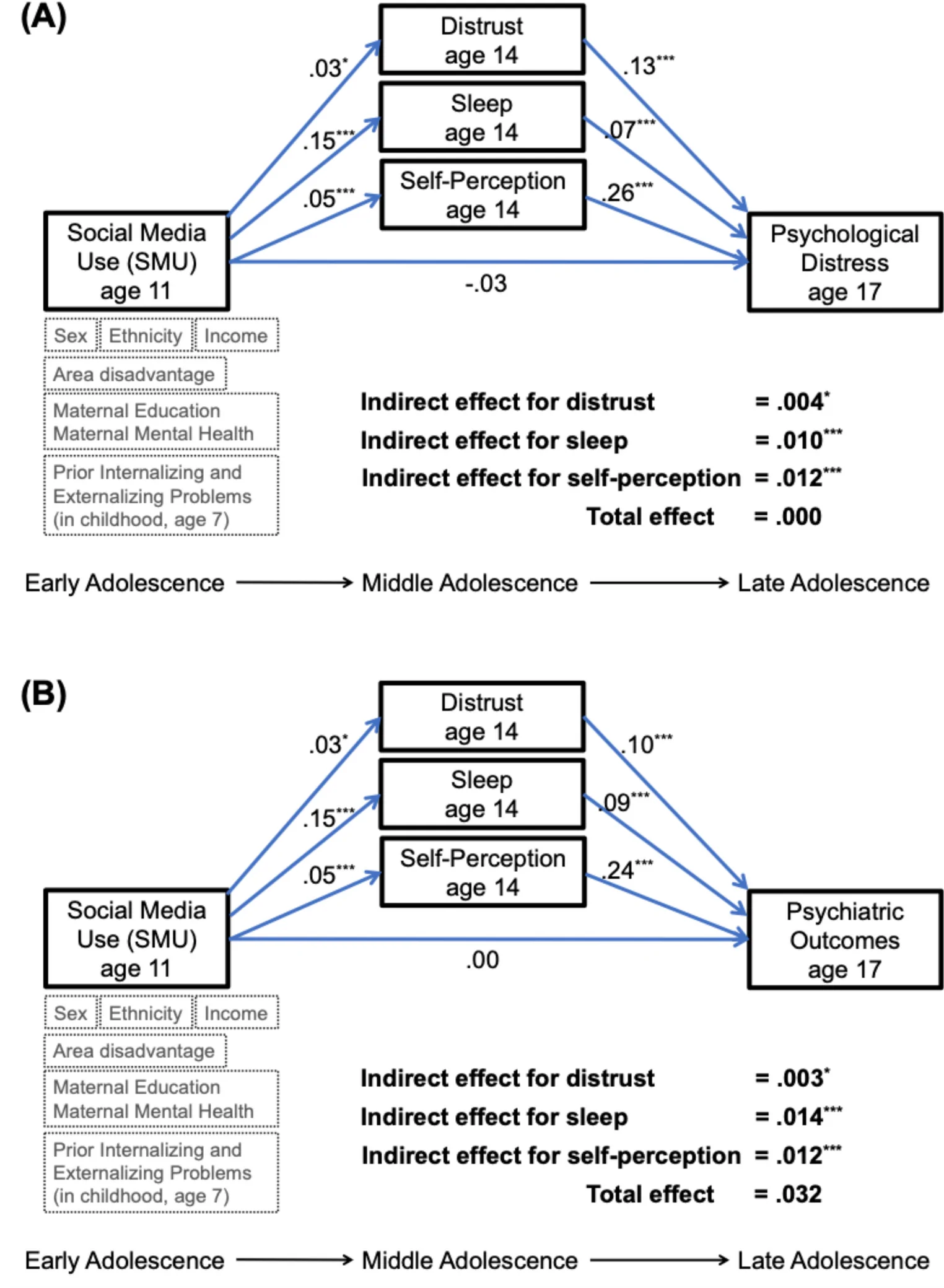
Model linking SMU to (**A**) psychological distress, and (**B**) psychiatric problems (depression/anxiety, self-harm, and suicidality) through distrust, time-to-sleep, and negative self-perception of physical appearance (standardized paths shown in fully adjusted models)

The demographics of participants in the age 11 wave of the survey can be found in Table 1, which also compares those included in the sample with those excluded from it. The sample of 8,913 participants was 52% female and 18% non-White. In terms of missingness in the sample, the highest amount occurred for participants’ prior mental health problems (age 7) with 790 missing values (9%). The mediator variables had between 7% (for time-to-sleep) and 8% (for self-perception) of missing data, while all other variables had only up to around 5% of their values missing [71]. Next, a correlation analysis (full results in the SOM [71]) showed that the highest positive correlation between confounders was for maternal psychological distress and the participants’ prior mental health difficulties at age 7, as well as between distrust and negative self-perception of physical appearance (* r=.30,p<.001* in both cases). The strongest negative correlation was between income and prior mental health at age 7 (* r=-.27,p<.001*). For the main analyses (Fig. 1 presents a simplified schematic of the models), we summarize results in separate subsections.

### Main analysis: psychological distress

There was a significant indirect association between SMU (age 11) and psychological distress (age 17) through distrust (age 14), $\:ab=.012,\:p=.020$, $\:95\%\:CI[0.002,\:0.022]$ (standardized $\:95\%\:CI[0.001,\:0.007]$), even after full adjustment (with sex, ethnicity, income, area disadvantage, maternal education and mental health, and the child’s prior mental health difficulties), as shown in Fig. 1. Time-to-sleep and negative self-perception were also significant mediators overall and in sex-stratified analyses (i.e. both for male and female cohort members), as shown in Table 2. The indirect association through distrust was significant for females ($\:ab=.019,\:p=.033$) but not males ($\:ab=.006,\:p>.05$). However, the total effect was close to zero and non-significant. These findings were replicated to a high level of accuracy in the sensitivity analysis with a different analytic sample [71].

### Secondary outcomes: Internalizing/externalizing problems

For internalizing (emotion and peer) problems, there was a significant indirect association between SMU and internalizing problems via distrust even after full adjustment, $\:ab=.010,\:p=.024$, $\:95\%\:CI\left[0.001,\:0.018\right]$ (standardized $\:95\%\:CI\left[0.001,\:0.008\right]$). As in the main analysis, the path via distrust was significant for females only, while the total effect was non-significant. However, in this case, the path through time-to-sleep was significant for males only (SOM [71], section ‘Additional 1A’).

Results for externalizing (conduct and hyperactivity/inattention) problems are summarised in Table 3. The findings on mediation effects reflect the same pattern as in the main analysis, however, the total effect was significant in this case, with standardized coefficient $\:0.071,\:p<.001,\:95\%\:CI\left[0.046,\:0.097\right]$. These findings remained robust in the sensitivity analysis [71].

### Secondary outcomes: psychiatric problems

For psychiatric problems (depression/anxiety diagnosis, self-harm, and suicidality), the direct effect of SMU was negligibly small (and non-significant), and the total effect was positive but non-significant (Fig. 1B). As before, there was full mediation via distrust for females only, with standardized $\:\alpha\:\beta\:=0.005,\:p=.041$, $\:95\%\:CI[0.001,\:0.010]$. Results for this case are summarized in Table 4. The findings were robust in the sensitivity analysis [71].

### Additional analysis: odds ratios for binary measures

Using binary measures on the complete data without survey weights, the crude odds ratio for psychopathology (Kessler-6 score > 12), given SMU exposure versus non-exposure, was $\:OR=1.25,\:p<.001,\:95\%CI\:[1.12,\:1.40].$ A diagnosis of depression or anxiety yielded $\:OR=1.44,\:p<.001,\:95\%CI\:\left[1.26,\:1.66\right],$ and similarly for self-harm, whereas the odds ratio for suicidality was considerably higher, $\:OR=1.75,\:p<.001,\:95\%CI\:\left[1.49,\:2.06\right]$. After adjusting for all confounders (but not the mediators), these ORs decreased and became non-significant except for suicidality, $\:OR=1.34,\:p=.002,\:95\%CI\:\left[1.11,\:1.61\right]$.

## Discussion

Our longitudinal analyses showed that social media use (SMU) in early adolescence (age 11) does not directly predict subsequent psychological distress or psychiatric problems (age 17) after adjusting for known confounders. Instead, we uncovered indirect associations between early-adolescent SMU and late-adolescent mental health difficulties through three mediators assessed in middle adolescence (age 14): interpersonal distrust, delayed bedtimes, and negative body image. Importantly, the pathway via interpersonal distrust was significant only among females, whereas delayed sleep and negative self-perception mediated risk equally in both sexes. These findings were recovered in a sensitivity analysis, and held true in additional analyses with secondary outcomes, namely, internalizing/externalizing problems, and a latent variable composed of clinical diagnosis of depression or anxiety, self-harm, and suicidality.

The finding of a female-specific mediation through distrust supports existing evidence that adolescent girls, who tend to show greater empathic concern and place a higher value on symmetrical reciprocity [73, 74], may be particularly susceptible to relationship-based stressors [75] in online contexts, where they often go for support [76]. Online interactions can instead provide fertile ground for social comparison, cyberbullying, and perceived exclusion [77–80]. Girls’ greater sensitivity to social evaluation [81] may heighten the impact of these negative experiences, eroding trust in others. Within the framework of Social Safety Theory, distrust and perceived social isolation or lack of social support may fester maladaptive social safety schemas, which play a key role in the pathogenesis or persistence of internalizing difficulties such as anxiety, depression, self-harm and suicidality over time, particularly in female adolescents [59].

Our findings on the role of sleep and negative self-image align with the evidence from previous cross-sectional studies [9, 10, 12, 29, 36]. Furthermore, the relatively weak prospective direct association between SMU and psychiatric outcomes in the general youth population confirms previous findings in the literature [12, 16, 25, 31, 35]. However, the mediating role of interpersonal distrust in these associations constitutes a novel pathway through which SMU degrades adolescent mental health. In addition, we showed that female adolescents are more susceptible to the impact of distrust than their male counterparts. To understand why this might be the case, we note that interpersonal distrust is an index of negative social safety schemas [56–59], that is, the perception of the social world as a hostile environment, rejecting, and lacking a sense of acceptance or belonging. Such schemas are known to foster anxiety and hypervigilance, which degrade physical and mental health over time through various biological mechanisms [57]. Therefore, our findings are in line with what would be expected by Social Safety Theory [56], and contribute to our expanding knowledge of the long-term impact of social media use on adolescents’ lives.

Finally, although our exposure measure indexed *frequency* rather than *type* of engagement with social media in early adolescence, distinctions between passive (e.g., scrolling, browsing) and active (e.g., posting, commenting) SMU may help interpret the indirect effects uncovered in the present work. Previous studies have provided evidence that passive use is more consistently linked to adverse outcomes via upward social comparison, appearance-focused self-evaluation, and perceived exclusion, whereas active, reciprocal exchanges can confer social support and a sense of belonging [82–85], although such effects are small, mixed, and context-dependent [31, 61]. In our study, the significant indirect pathways through later bedtimes and negative self-image are compatible with mechanisms commonly implicated in passive consumption (i.e., late-night, noninteractive scrolling that delays sleep, and repeated exposure to idealized content that amplifies appearance-based comparison). At the same time, active use is not uniformly protective. Certain active behaviors—such as public posting with contingent feedback or co-rumination in private chats—may heighten evaluation concerns and reinforce negative affect, potentially sustaining distrust for some adolescents. Such tentative interpretations of our findings underscore that ‘how’ adolescents engage with social media may be at least as important as ‘how much’ [86] ^86^.

### Limitations and future directions

The findings of this study should be considered in the context of their limitations. First, the measures used were based on self-reports. Although the importance and relevance of self-disclosure is not in question, it must be acknowledged that it poses a limitation in terms of the results reported. Second, SMU exposure at age 11 was a single item asking participants about the frequency of their use of social media, and the mediator of interpersonal distrust at age 14 also was a single item. In both cases, it would have been beneficial to have broader, multi-item scales, providing more detailed information about SMU and distrust. Third, recent longitudinal studies have provided emerging evidence of a bidirectional association between SMU and adolescent mental health [29, 82, 87, 88]—a key limitation of the present work is that bidirectionality could not be tested. Fourth, the analysis relied on data that covered a 10-year period (ages 7 to 17 years), but there were only four survey waves in that period—more detailed, granular data would have been beneficial. Fifth, the analysis relied entirely on observational birth cohort data, without any experimental manipulation of the exposure or mediating variables, therefore no strict causal claims can be made (as residual confounding cannot be ruled out), even though we used correctly ordered variables in a suitable structural equation model. Finally, we did not adjust for baseline levels of the mediators, as these were not available in the survey data at age 11; failing to include such autoregressive paths may conflate stable, trait-like differences with within-person change [89]. Therefore, our indirect paths should be interpreted as associations consistent with mediation rather than definitive causal effects.

Future research should address these limitations by using independent clinician reports for the outcomes and more in-depth instruments for the key variables (to measure interpersonal trust, and to distinguish between active and passive use of social media); by investigating in detail the possibility of reverse-causality and bidirectional associations (e.g., that low self-esteem and negative perceptions of physical appearance precede problematic SMU [90]); and, finally, by experimentally manipulating interpersonal trust under conditions of controlled exposure to social media in a laboratory setting.

### Conclusions and implications for public health

The present study shows that distrust, later sleep times, and negative body image, fully mediate the association between exposure to social media in early adolescence and psychiatric outcomes in late adolescence. The indirect pathway through interpersonal distrust impacts female adolescents more than males. However, the direct prospective association between social media use in early adolescence and psychological distress in late adolescence was small and non-significant in this large, nationally representative birth cohort from the United Kingdom. These findings suggest that interventions in early and middle adolescence aimed at fostering a sense of trust and social safety [59, 91] (positive perceptions of social support and belonging) can help mitigate the small but significant impact of social media use on mental health in the long term, especially for adolescent girls. Interventions for good sleep hygiene [92] and positive body image [93] should also be considered in this respect for both adolescent boys and girls.

**Table 1:** Demographic characteristics of the sample at the age 11 wave, and a bias analysis (unweighted) between those included in the final sample and those excluded from it

Characteristic	Excluded from sample, *N* = 3,819	Analytic sample, *N* = 8,913	*p* ^1^
**Sex**, n (%)			< 0.001
*Male*	2,133 (56)	4,294 (48)	
*Female*	1,686 (44)	4,619 (52)	
**Ethnicity**, n (%)			< 0.001
*White*	3,247 (86)	7,271 (82)	
*Mixed*	109 (2.9)	246 (2.8)	
*Indian*	68 (1.8)	258 (2.9)	
*Pakistani and Bangladeshi*	213 (5.6)	667 (7.5)	
*Black or Black British*	121 (3.2)	289 (3.3)	
*Other Ethnic group*	38 (0.9)	141 (1.4)	
*(Missing)*	23	41	
**Area disadvantage**, n (%)			< 0.001
*England - Advantaged*	901 (24)	2,571 (29)	
*England - Disadvantaged*	1,003 (26)	2,129 (24)	
*England - Ethnic*	437 (11)	1,182 (13)	
*Wales - Advantaged*	165 (4.3)	414 (4.6)	
*Wales - Disadvantaged*	416 (11)	819 (9.2)	
*Scotland - Advantaged*	218 (5.7)	534 (6.0)	
*Scotland - Disadvantaged*	252 (6.6)	427 (4.8)	
*Northern Ireland - Advantaged*	134 (3.5)	355 (4.0)	
*Northern Ireland - Disadvantaged*	293 (7.7)	482 (5.4)	
**Income**, Mean (SD)	2.72 (1.35)	3.21 (1.39)	< 0.001
*(in quintiles)*	*n (%)*	*n (%)*	
*1 (lowest)*	926 (24)	1,425 (16)	
*2*	894 (23)	1,492 (17)	
*3*	811 (21)	1,878 (21)	
*4*	705 (18)	2,058 (23)	
*5 (highest)*	483 (13)	2,060 (23)	
**Maternal education** (degree), n (%)	166 (4.5)	760 (8.8)	< 0.001
*(Missing)*	134	293	
**Maternal distress**, Mean (SD)	4.4 (4.8)	3.8 (4.3)	< 0.001
*(Missing)*	235	480	
**Prior mental health difficulties** (age 7), Mean (SD)	8.2 (5.7)	7.0 (5.2)	< 0.001
*(Missing)*	680	790	
**Psychological distress** (age 17), Mean (SD)	7.5 (5.1)	7.3 (4.9)	0.62
*(Missing)*	3,625	0	
**Distrust** (age 14), Mean (SD)	3.87 (2.19)	3.82 (2.17)	0.39
*(Missing)*	1,777	607	
**Social media use** (age 11), Mean (SD)	1.68 (1.74)	1.25 (1.64)	< 0.001
*(in frequency of use)*	*n (%)*	*n (%)*	
*0 (never)*	1,623 (47)	5,243 (59)	
*1*	195 (5.7)	454 (5.1)	
*2*	209 (6.1)	517 (5.8)	
*3*	526 (15)	1,126 (13)	
*4 (most days)*	897 (26)	1,573 (18)	
*(Missing)*	369	0	
**Total mental health problems** (age 17), Mean (SD)	13.0 (5.8)	12.2 (5.6)	0.039
*(Missing)*	3,604	166	
**Internalizing problems** (age 17), Mean (SD)	6.1 (3.5)	5.6 (3.5)	0.050
*(Missing)*	3,603	166	
**Externalizing problems** (age 17), Mean (SD)	6.0 (3.3)	5.6 (3.3)	0.12
*(Missing)*	3,604	166	
**Time-to-sleep** (age 14), Mean (SD)	3.02 (0.99)	2.93 (0.94)	< 0.001
*(Hour slot)*	*n (%)*	*n (%)*	< 0.001
*1 (Before 9pm)*	102 (5.0)	366 (4.4)	
*2 (9pm to 10pm)*	529 (26)	2,452 (29)	
*3 (10pm to 11pm)*	827 (40)	3,383 (41)	
*4 (11pm to midnight)*	436 (21)	1,657 (20)	
*5 (After midnight)*	166 (8.1)	460 (5.5)	
*(Missing)*	1,759	595	
**Self-perception** (age 14), n (%)	3.16 (1.60)	3.21 (1.56)	0.092
*(Missing)*	1,828	673	
**Diagnosis of depression or anxiety** (age 17), n (%)			0.23
*0 (No)*	194 (87)	7,984 (90)	
*1 (Yes)*	29 (13)	917 (10)	
*(Missing)*	3,596	12	
**Self-harm** (age 17), n (%)			0.18
*0 (No)*	176 (82)	6,792 (78)	
*1 (Yes)*	39 (18)	1,942 (22)	
*(Missing)*	3,604	179	
**Suicide attempt** (age 17), n (%)			0.38
*0 (No)*	204 (94)	8,084 (93)	
*1 (Yes)*	12 (5.6)	642 (7.4)	
*(Missing)*	3,603	187	

^*1*^Pearson’s Chi-squared test (categorical variables) or Welch Two Sample t-test (numerical variables) | Note: Further details on variable frequencies and Millennium Cohort Study labels can be found in the Supplementary Online Material (SOM) [71]^71^.

**Table 2:** Survey-weighted, imputed, adjusted structural equation model (main analysis: psychological distress, Kessler-6 scale) for all participants, females, and males. Showing unstandardized coefficients (standard errors) for the full analytic sample ($\:N=\mathrm{8,913}$)

	All (*N* = 8,913)	Females (*N* = 4,619)	Males (*N* = 4,294)
	*Regression Slopes* – Estimate (Std. Err.)		
*Psychological distress (age 17)*			
Social media use (age 11)	−0.08(0.04)	−0.05(0.06)	−0.13(0.05)^*^
Distrust (age 14)	0.31(0.03)^***^	0.36(0.04)^***^	0.24(0.04)^***^
Time-to-sleep (age 14)	0.35(0.07)^***^	0.45(0.10)^***^	0.27(0.08)^**^
Self-perception (age 14)	0.82(0.04)^***^	0.81(0.06)^***^	0.79(0.06)^***^
Sex: Male	−1.41(0.11)^***^		
Income	0.02(0.06)	−0.03(0.09)	0.07(0.08)
Maternal education	0.10(0.20)	−0.19(0.26)	0.39(0.29)
Black or Black British	−0.35(0.38)	−0.13(0.47)	−0.56(0.50)
Indian	−0.75(0.39)	−0.61(0.53)	−0.76(0.57)
Mixed	−0.00(0.33)	−0.34(0.50)	0.30(0.45)
Other Ethnic Group	0.26(0.42)	0.67(0.66)	−0.24(0.55)
Pakistani & Bangladeshi	0.98(1.33)	0.11(1.83)	2.77(1.57)
England - Disadvantaged	−0.26(0.17)	−0.31(0.26)	−0.19(0.21)
England - Ethnic	−0.59(0.24)^*^	−0.56(0.33)	−0.62(0.30)^*^
Northern Ireland -Advantaged	−0.31(0.24)	−0.43(0.35)	−0.21(0.38)
Northern Ireland -Disadvantaged	−0.31(0.24)	−0.66(0.35)	0.07(0.33)
Scotland - Advantaged	0.49(0.23)^*^	0.57(0.35)	0.45(0.29)
Scotland - Disadvantaged	0.19(0.24)	−0.33(0.39)	0.75(0.28)^**^
Wales - Advantaged	0.03(0.23)	0.03(0.34)	0.12(0.32)
Wales - Disadvantaged	0.21(0.22)	0.54(0.33)	−0.07(0.28)
Maternal distress	0.06(0.02)^**^	0.09(0.03)^**^	0.01(0.02)
Prior mental health difficulties (age 7)	0.07(0.01)^***^	0.09(0.02)^***^	0.06(0.02)^***^
*Distrust (age 14)*			
Social media use (age 11)	0.04(0.02)^*^	0.05(0.02)^*^	0.02(0.02)
*(confounders)* ^+^			
*Time-to-sleep (age 14)*			
Social media use (age 11)	0.09(0.01)^***^	0.09(0.01)^***^	0.09(0.01)^***^
*(confounders)* ^+^			
*Self-perception (age 14)*			
Social media use (age 11)	0.05(0.01)^***^	0.05(0.01)^**^	0.04(0.02)^**^
*(confounders)* ^+^			
	*Indirect Paths*		
*a.b*_*1*_ (through distrust)	0.01(0.00)^*^	0.02(0.01)^*^	0.01(0.01)
*a.b*_*2*_ (time-to-sleep)	0.03(0.01)^***^	0.04(0.01)^***^	0.02(0.01)^**^
*a.b*_*3*_ (self-perception)	0.04(0.01)^***^	0.04(0.01)^**^	0.03(0.01)^**^
	*Total Effect*		
*Total*	0.00(0.05)	0.05(0.07)	−0.06(0.05)

^*^p < 0.05, ^**^p < 0.01, ^***^p < 0.001 | ^+^Estimates for confounder coefficients are shown only for the outcome (psychological distress scale); consult the SOM [71] for all coefficient estimates (with standard errors and p-values) of confounding variables in the exposure-mediator relationships (i.e., SMU’s associations with distrust, time-to-sleep, and self-perception).

**Table 3:** Survey-weighted, imputed, adjusted structural equation model (additional analysis: externalizing problems) for all participants, females, and males. Showing unstandardized coefficients (standard errors) for the full analytic sample ($\:N=\mathrm{8,913}$)

	All (*N* = 8,913)	Females (*N* = 4,619)	Males (*N* = 4,294)
	*Regression Slopes* – Estimate (Std. Err.)		
*Externalizing Problems (age 17)*			
Social media use (age 11)	0.09(0.03)^***^	0.10(0.03)^**^	0.07(0.04)
Distrust (age 14)	0.17(0.02)^***^	0.23(0.03)^***^	0.10(0.03)^***^
Time-to-sleep (age 14)	0.36(0.05)^***^	0.37(0.07)^***^	0.36(0.07)^***^
Self-perception (age 14)	0.30(0.03)^***^	0.25(0.04)^***^	0.36(0.04)^***^
Sex: Male	0.79(0.09)^***^		
*(confounders)* ^+^			
*Distrust (age 14)*			
Social media use (age 11)	0.04(0.02)^*^	0.05(0.02)^*^	0.02(0.02)
*(confounders)* ^+^			
*Time-to-sleep (age 14)*			
Social media use (age 11)	0.09(0.01)^***^	0.09(0.01)^***^	0.09(0.01)^***^
*(confounders)* ^+^			
*Self-perception (age 14)*			
Social media use (age 11)	0.05(0.01)^***^	0.05(0.02)^**^	0.04(0.02)^**^
*(confounders)* ^+^			
	*Indirect Paths*		
*a.b*_*1*_ (through distrust)	0.01(0.00)^*^	0.01(0.01)^*^	0.00(0.00)
*a.b*_*2*_ (time-to-sleep)	0.03(0.01)^***^	0.04(0.01)^***^	0.03(0.01)^***^
*a.b*_*3*_ (self-perception)	0.01(0.00)^***^	0.01(0.01)^**^	0.02(0.01)^*^
	*Total Effect*		
*Total*	0.15(0.03)^***^	0.16(0.03)^***^	0.12(0.04)^**^

^*^p < 0.05, ^**^p < 0.01, ^***^p < 0.001 | ^+^Estimates for all confounder coefficients (with standard errors and p-values) can be found in the SOM [71].

**Table 4:** Survey-weighted, imputed, adjusted structural equation model (additional analysis: latent variable of ‘psychiatric problems’) for all participants, females, and males. Showing unstandardized coefficients (standard errors) for the full analytic sample ($\:N=\mathrm{8,913}$)

	All (*N* = 8,913)	Females (*N* = 4,619)	Males (*N* = 4,294)
	*Loadings*		
Depression/anxiety	1	1	1
Suicide attempt	1.04(0.05)^***^	1.03(0.07)^***^	1.22(0.13)^***^
Self-harm	1.29(0.07)^***^	1.21(0.08)^***^	1.49(0.17)^***^
	*Regression Slopes* – Estimate (Std. Err.)		
*Psychiatric Problems (latent variable*,* age 17)*			
Social media use (age 11)	0.00(0.00)	0.00(0.01)	−0.00(0.00)
Distrust (age 14)	0.01(0.00)^***^	0.01(0.00)^***^	0.00(0.00)^*^
Time-to-sleep (age 14)	0.02(0.00)^***^	0.02(0.01)^***^	0.01(0.00)^*^
Self-perception (age 14)	0.03(0.00)^***^	0.03(0.00)^***^	0.02(0.00)^***^
Male	−0.04(0.01)^***^		
*(confounders)* ^+^			
*Distrust (age 14)*			
Social media use (age 11)	0.04(0.02)^*^	0.05(0.02)^*^	0.02(0.02)
*(confounders)* ^+^			
*Time-to-sleep (age 14)*			
Social media use (age 11)	0.09(0.01)^***^	0.09(0.01)^***^	0.09(0.01)^***^
*(confounders)* ^+^			
*Self-perception (age 14)*			
Social media use (age 11)	0.05(0.01)^***^	0.05(0.02)^**^	0.04(0.02)^**^
*(confounders)* ^+^			
	*Indirect Paths*		
*a.b*_*1*_ (through distrust)	0.000(0.00)^*^	0.001(0.00)^*^	0.000(0.00)
*a.b*_*2*_ (time-to-sleep)	0.002(0.00)^***^	0.002(0.00)^***^	0.001(0.00)^*^
*a.b*_*3*_ (self-perception)	0.001(0.00)^***^	0.002(0.00)^**^	0.001(0.00)^*^
	*Total Effect*		
*Total*	0.004(0.00)	0.006(0.00)^*^	0.000(0.00)
	*Fit Indices* ^^^		
RMSEA	0.02	0.02	0.02
SRMR	0.01	0.01	0.01
CFI	0.98	0.97	0.97
TLI	0.93	0.92	0.91
Scaled χ^2^	162.95(44)^***^	117.97(42)^***^	73.19(42)^***^

^*^p < 0.05, ^**^p < 0.01, ^***^p < 0.001 | ^+^Estimates for all confounder coefficients (with standard errors and p-values) can be found in the SOM [71]. | ^^^RMSEA = Root Mean Square Error of Approximation; SRMR = Standardized Root Mean Square Residual; CFI = Comparative Fit Index; TLI = Tucker-Lewis Index.

## Data Availability

Supplemental Online Material (SOM) has been published on the Open Science Framework repository; the supplementary document contains the entire output of the code in R, which was used to produce the results of this study. It can be accessed via: https://osf.io/hgbz8.

## Footnotes

1 Given the focus of this study, a different choice of inclusion criteria is also possible, i.e., that participants have valid records in the exposure (SMU) and the focal mediator (distrust). This leads to a larger analytic sample, with *N* = 10,112 (51% female), but the missingness is around 17%-19% for psychiatric outcomes. Therefore, in a sensitivity analysis, this larger sample is used instead to assess its impact on the results (after data imputation).
